# Strong agreement between self-administered and interview-obtained bowel function score in patients with Hirschsprung disease and anorectal malformation

**DOI:** 10.1007/s00383-025-06207-3

**Published:** 2025-10-06

**Authors:** Remi Andre Karlsen, Anders Telle Hoel, Kristin Bjørnland

**Affiliations:** 1 Institute of Clinical Medicine, University of Oslo, Oslo, Norway; 2 Department of Pediatric Surgery, Oslo University Hospital, Oslo, Norway

**Keywords:** Hirschsprung, Hirschsprung disease, Anorectal malformation, Bowel function score, Bfs, Constipation, Fecal incontinence, Incontinence, Postoperative bowel function

## Abstract

**Background:**

The Bowel Function Score (BFS) questionnaire is the most widely utilized tool for assessing bowel function in patients with Hirschsprung disease (HD) or anorectal malformation (ARM). However, the questionnaire has not undergone a formal validation process. This study aimed to compare self-administered responses with those obtained during clinical consultations to determine whether patients can reliably report their bowel function when completing the questionnaire independently.

**Methods:**

"Patients with HD or ARM and/or with their parents, completed the BFS questionnaire prior to their outpatient clinic visit. During consultations, the questionnaires were reviewed, with any missing or unclear responses addressed and adjustments recorded. The agreement between self-administered and interview-obtained BFS scores was assessed using the intraclass correlation coefficient (ICC)."

**Results:**

A total of 103 questionnaires with 721 answered questions were evaluated. The agreement between self-administered and interview-based BFS scores was found to be nearly perfect (ICC) 0.96. The question recording frequency of defecation, was the only item that did not demonstrate perfect agreement.

**Conclusion:**

This study supports the use of the self-administered BFS questionnaire as a reliable tool for assessing bowel function in HD and ARM patients in both clinical practice and research.

## Introduction

Hirschsprung disease (HD) and anorectal malformation (ARM) are rare congenital conditions that affect approximately one out of 5000 children [1, 2]. HD is characterized by absence of ganglion cells in the distal bowel, while ARM includes a spectrum of congenital anomalies in the anorectal area. Long-term bowel problems are common in both ARM and HD patients after reconstructive surgery. In HD patients, fecal incontinence and constipation occur in 3–48% and 1–56%, respectively [3], while up to 80% of ARM patients experience fecal incontinence and/or constipation [4]. Precise evaluation and monitoring of bowel function is important for healthcare providers to identify problems, institute and adjust treatment. Use of a bowel function questionnaire can be of great help in evaluating bowel function, and use of such questionnaires in clinical practice has been shown to improve patient outcomes [5]. Moreover, standardized recording of postoperative bowel function is essential in clinical research for comparing bowel function across different interventions and patient populations.

**Fig. 1:**
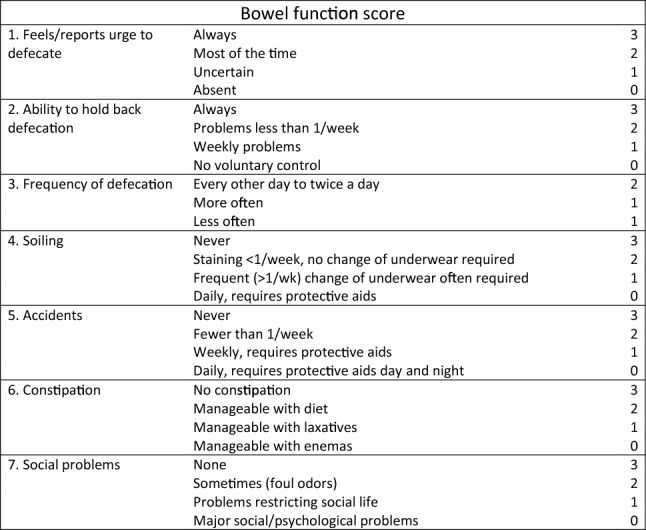
Bowel function score [8]

The most frequently used instrument for assessing bowel function in HD and ARM patients, is the Bowel Function Score (BFS) questionnaire developed by Rintala and Lindahl [6, 7]. It was originally designed for assessment of bowel function in ARM patients and records elements such as ability to hold back defecation, sensation, frequency of defecation, incontinence, constipation, and how bowel function impacts social life (Fig. 1). The BFS questionnaire lacks formal validation, and there are no published reports on translation or validation in other languages. There are, however, available data from a large Finnish population of healthy controls aged 4–26 years [8].

Answers to a questionnaire can be influenced by several factors, including the respondent, the mode of administration, and the setting. It has been shown that patients report more favorable outcomes during telephone interviews compared to online or paper-based surveys [8]. In addition, patients may answer in a more positive way when a doctor is present [9]. Consequently, answers to bowel function questionnaires may be influenced by several factors. In adults, strong agreement has been shown for patient- and interviewer based scores for St Marks and Wexner score bowel function questionnaires [10]. The BFS questionnaire is the most widely used questionnaire in studies on bowel function in HD and ARM patients [6]. In these studies the questionnaire has been administered in different ways. Since there is no information on the agreement between self-administered and interview-based answers to the BFS questionnaire, we compared self-administered answers to answers obtained during a clinical consultation.

## Methods

### Patients and data collection

Patients with HD or ARM, 4–18 years old, were asked to complete the BFS questionnaire while awaiting their appointment in the outpatient clinic. Depending on age and intellectual capacity of the patient, the BFS questionnaire was answered by the patient alone, by patient and parent together, or only by the parent(s). A paper version of the questionnaire was handed out without any specific instructions. During the clinical consultation, answers to the various questions in the BFS questionnaire were discussed with the patients and/or parents. If any question had a missing answer, an answer was recorded. Any unclear answer was corrected. The personnel who conducted the interviews were members of the patients’ care team, but they were not the surgeons who had operated on the patients.

### Translation and scoring of the Bowel Function Score questionnaire

The BFS questionnaire was developed in Finland which has two official languages (Finnish and Swedish) [7]. The questionnaire was translated from Swedish to Norwegian by the authors. Due to great similarities between Swedish and Norwegian, no forward and backward translations were performed.

Scores from completed questionnaires were calculated as outlined in the original paper (6). When encountering unclear markings in the self-administered questionnaire, the average of the closest answers was used to calculate self-administered scores. During the consultation, the answers to the BFS questionnaire were discussed with either a research fellow, a stoma nurse or a pediatric surgeon who made the interview-based score at the end of the consultation.

### Definitions

A questionnaire was defined as “complete” if all questions were answered with a single marking. An “unclear” marking was defined as two markings to the same question or a marking between two answers. A “corrected” answer refers to any response that was adjusted after the consultation.

### Statistics

To calculate the level of agreement between self-administered and interview-obtained scores we used the intraclass correlation coefficient (ICC), 1 implying perfect agreement and 0 none. The level of agreement between self-administered and interview-obtained answers for individual questions was assessed with quadratic weighted kappa statistics for ordinal level variables. The agreement was rated slight (κ ≤ 0.2), fair (0.2 < κ ≤ 0.4), moderate (0.4 < κ ≤ 0.6), substantial (0.6 < κ ≤ 0.8), or almost perfect (0.8 < κ ≤ 1). Numerical variables are presented as median with range (min–max) for not normally distributed data and as mean with standard deviation (SD) for normally distributed data. Normality was assessed by the Shapiro–Wilk-test in combination with visual assessment of histograms and normality plots. T-test or Mann–Whitney *U* test were used as appropriate. Chi-square test or Fisher’s exact test were used to compare categorical variables. Spearman's rank correlation coefficient was used for bivariate correlation analysis. Statistical significance was set at p < 0.05. Statistical analysis was performed using SPSS statistics 29 (IBM Corp. Armonk, NY).

### Ethics

The study was approved by the regional committee for medical and health research ethics (2018/2009) and the Data Protection Officers at Oslo university hospital (2017/4913).

## Results

In total 103 questionnaires, including 721 questions, were assessed, and approximately half were answered by patient and parent(s) together (Table 1). Eight patients answered the questionnaire twice, and none answered more than two questionnaires. Of the 721 questions, 24 (3%) had unclear markings, and 6 (0.7%) were missing. Question #4 recording soiling had the highest number of unclear markings and corrected answers, whereas question #7 about social problems had the fewest (Table 2).

**Table 1:** Bowel Function Score questionnaires answered by patients and/or parents with Hirschsprung disease (HD) or Anorectal malformations (ARM)

	All (N = 103)	ARM (N = 54)	HD (N = 49)	p-values^c^
Age (years)	9 (4–18)	8 (4–18)	10 (4–18)	0.04
Sex (boys)	68 (66%)	29 (54%)	39 (80%)	< 0.01
Questionnaire answered by				
Patient alone	17 (17%)	7 (13%)	10 (20%)	0.22
Parent alone	38 (37%)	24 (44%)	14 (29%)	
Patient and parent together	48 (47%)	23 (43%)	25 (51%)	
Completed questionnaires^a^	82 (80%)	42 (79%)	40 (82%)	0.63
Questionnaires with				
Missing answers	6 (6%)	2 (4%)	4 (8%)	0.29
Unclear markings^b^	18 (17%)	11 (20%)	7 (14%)	0.66

^a^Questionnaires with all questions answered with a single marking by the patient and/or parent

^b^A question answered with two markings or a marking between two answers

^c^Comparing numbers between HD and ARM patients

**Table 2:** Number of missing answers, unclear markings, and corrected answers to individual questions in the Bowel Function Score questionnaire

Question	Missing answers	Unclear markings	Corrected answers	Intradyad difference	Kappa
#1	0	3	13	0 ((−1) −3)	0.83
#2	3	5	15	0 ((−0,5) −1)	0.95
#3	0	1	13	0 ((−1) −1)	0.71
#4	0	6	17	0 ((−2) −1.5)	0.89
#5	0	2	16	0 ((−3) −2)	0.81
#6	2	5	16	0 (−3) −3	0.82
#7	1	1	1	0 ((−0,5) −0)	0.99

Intradyad difference between self-administered and interview-obtained answers is reported, as well as the level of agreement calculated with quadratic weighted kappa. A total of 103 questionnaires were answered

There was almost perfect agreement between self-administered and interview-based BFS scores (ICC 0.96; Table 3). Only question #3 recording frequency of defecation did not show perfect agreement by kappa statistics.

**Table 3:** Bowel function scores reported by patients and/or parents^a^ and by healthcare providers after interview^b^

	N	Self-administered score^a^	Interview-obtained score^b^	Intradyad difference	ICC
Total score					
Overall	98	16 (4–20)	16 (3–20)	0 ((−4) −5)	0.96
Arm	52	15 (4–20)	15 (3–20)	0 ((−4) −5)	0.96
HD	46	16 (7–20)	16 (7–20)	0 ((−3) −3)	0.97

There was no significant difference in frequency of missing or unclear markings between questionnaires answered by ARM or HD patients. There was, however, a difference in the frequency of corrected answers between the two patient populations as 36 (69%) of questionnaires answered by ARM patients had one or more corrections compared to 21 (46%) of the HD questionnaires (p = 0.02). There was no correlation between the number of corrected answers and patient scores (p = 0.5), sex of the patient (p = 0.6), or if the questionnaire was answered by the patient alone, together with parent(s), or only by parent(s) (p = 0.9).

## Discussion

In this study we found strong agreement between self-administered and interview-based BFS scores. Even though use of validated questionnaires is the gold standard in research, our study shows that the BFS questionnaire can be a good option for assessment of bowel function in HD and ARM patients. Our findings support a recently published systematic review listing BFS as one of three recommended bowel function questionnaires for use in HD patients, and it is the preferred bowel function questionnaire of the OASIS-Holistic Care in Hirschsprung Disease Network Group [6, 11].

Question #3 recording frequency of defecation was the only question without perfect agreement between self-administered and interview-obtained answers. This question differs slightly from the others, as the answering options do not follow the same ordinal structure of four levels indicating increasing severity of problems as the rest of the questions. The clinical consultation revealed that some misunderstood the answering alternatives, not realizing that the first option was an interval. If the answers had been re-phrased to “less often than every other day,” “every other day to twice a day,” and “more often than twice a day” the question would probably have been easier to understand. However, this would break the structure in which the most favorable response always appears first. Therefore, a brief explanation or added note to question #3 is suggested.

Unclear markings are impossible to assign a definitive score without speaking directly to the patient. Unclear markings may be unintentional or because it is difficult to choose a single answer. When talking to the patients and parents, the latter seemed to be the most likely explanation. Symptoms and bowel function can fluctuate, and it may therefore be challenging to select one answer that accurately reflects the overall experience. Question #4, which concerns soiling, had the highest number of unclear markings. Together with question #5, it addresses both frequency and consequences of soiling and fecal accidents. This dual focus can make answering more difficult. For example, infrequent soiling does not necessarily mean that a change of underwear is never needed, just as daily soiling does not always require the use of protective aids. Some individuals may choose to use protection even when soiling episodes are rare, while others may not use protective aids even if they soil every day if the soiling is minimal.

We found that answers from the ARM group were corrected more often than those from the HD group. This was surprising since the questionnaire was originally developed to assess bowel function in ARM patients. We speculate that bowel function in ARM patients may be more irregular than in HD patients, which could make it harder for them to answer consistently. Since the BFS questionnaire was developed for ARM patients, it does not address obstructive bowel problems, a problem frequently occurring in HD patients. It is therefore possible that obstructive problems are missed when the BFS is used. It could also be that obstructive problems are reported as constipation and therefore are captured by question #6. Lastly, obstructive problems are often more pronounced in the first period after the pull-through and patients tend to overcome these problems as they get older. Most of the patients in this study had undergone pull-through many years ago and obstructive problems were therefore not prominent. Since severity of symptoms is graded by treatment intensity, adding botulinum toxin injections to grade 3 could make the response more precise for patients needing botulinum toxin injections, but not regular enemas.

In conclusion, we found strong agreement between self-administered and interview-based BFS scores. Our findings support the frequent use of the BFS questionnaire in HD and ARM research as it is a short questionnaire accurately describing bowel function.

## Data Availability

No datasets were generated or analysed during the current study.
